# Contrast-enhanced ultrasound improves the potency of fine-needle aspiration in thyroid nodules with high inadequate risk

**DOI:** 10.1186/s12880-022-00805-6

**Published:** 2022-05-02

**Authors:** Tinghui Yin, Bowen Zheng, Yufan Lian, Haifeng Li, Lei Tan, Shicheng Xu, Yong Liu, Tao Wu, Jie Ren

**Affiliations:** 1 Department of Medical Ultrasonic, Laboratory of Novel Optoacoustic (Ultrasonic) Imaging, The Third Affiliated Hospital of Sun Yat-Sen University, Guangzhou, 510630 China; 2 Department of Pathology, The Third Affiliated Hospital of Sun Yat-Sen University, Guangzhou, 510630 China

**Keywords:** Contrast-enhanced ultrasound, Thyroid nodules, Fine-needle aspiration, Biopsy, Adequacy

## Abstract

**Background:**

This study aims to determine the clinical value of contrast enhanced ultrasound (CEUS) for fine-needle aspiration (FNA) of high inadequate risky thyroid nodules.

**Methods:**

During April 2018 and April 2021, consecutive 3748 thyroid nodules underwent FNA were retrospectively analyzed. CEUS guided FNA (CEUS-FNA) was applied in 115 nodules with high inadequate risk in Lingnan Campus. Ten nodules underwent CEUS-FNA presented non-enhancing, and would be further analyzed independently. Other 105 partial or total enhancing nodules were included as CEUS-FNA group, and 210 nodules with high inadequate risk in Tianhe Campus were match as the US-FNA control group. FNA specimens were collected for liquid-based preparation. Cytological results were classified following the Bethesda System for Reporting Thyroid Cytopathology.

**Results:**

The overall FNA specimen inadequate rate in our center was 6.6%. All of the ten non-enhancing nodules under CEUS have an inadequate result in cytopathological analyzes. The subsequent postoperative pathology and follow-up ultrasonography showed the non-enhancing nodules were benign or stable without further malignant features. Total specimen inadequate rate of high inadequate risk thyroid nodules in CEUS-FNA group was significantly lower than US-FNA group (6.7% vs. 16.7%, *P* = 0.014). Further stratified analyzed shown that FNA under US guidance, the inadequate rates in cystic, predominantly cystic, predominantly solid and solid sub-groups were 28.1%, 17.1%, 10.0% and 9.2% (*P* = 0.019). In contrast, the inadequate rates in cystic, predominantly cystic, predominantly solid and solid sub-groups were 7.4%, 6.7%, 5.6% and 6.7% (*P* = 0.996) in CEUS-FNA group.

**Conclusions:**

CEUS can improve the specimen adequacy of FNA in high inadequate risk thyroid nodules by avoiding unnecessary FNAs of the non-enhancing nodules, and accurately locating the viable tissue and precise guidance in real-time. CEUS is a recommend modality for FNA guidance of high inadequate risk thyroid nodules.

## Background

With the strengthening of healthcare awareness of people and continuous improvement of ultrasonic imaging techniques, the detection rate of thyroid nodules, especially small nodules, is raising rapidly in the recent decades. However, the high incidence and the overtreatment of the thyroid nodules are now growing public health concerns [1]. Ultrasound (US)-guided fine-needle aspiration (US-FNA) biopsy is a simple, effective and reliable technique for telling the benignancy or malignancy of thyroid nodules [2], and also has been shown to reduce the number of thyroidectomies and overall medical care cost [1]. Obtaining sufficient specimen from the nodules is the prerequisite for accurate cytological diagnosis after US-FNA. However, the current reports on adequacy of thyroid US-FNA are quite different (inadequate rate: 3–38%) [3]. There is a critical need to improve the specimen adequate rate for superior diagnostic efficacy of FNA.

It is reported that composition of thyroid nodules has a definite effect on acquiring specimen during US-FNA. Thyroid nodules with high cystic component [4, 5], hemorrhage or with macrocalcification [6] have higher risk of specimen inadequacy [7]. Meanwhile, solid nodule undergo reduction in size, which is known as degenerating nodules with malignant US features, often result in lower specimen acquisition [8, 9]. The high-risk factors (cystic, nodules with hemorrhage and suspicious degenerating nodules) may reduce the specimen acquisition during US-FNA by the following principles: (1) viable tissues are rare in cystic nodules; (2) conventional ultrasound has difficulty in distinguish viable tissues from the inactive tissues. Therefore, we hypothesize that the successful puncture of the viable tissue in high inadequate risky thyroid nodules, is a key factor in reducing the cytological inadequate rate of FNA.

Contrast-enhanced ultrasound (CEUS) is a safe, convenient and cost-effective imaging modality, and approved for thyroid imaging [10–12]. Clinical evidence has confirmed that CEUS detects viable tissue by visualizing microvascular perfusion [13]. Consequently, using CEUS to detect and locate the survival tissue of the thyroid nodule for puncturing guidance, may possibly increase inadequacy in FNA of high inadequate risky thyroid nodules. The objective of this study was to retrospectively analyze the specimen adequacy of CEUS guided FNA (CEUS-FNA) and US-FNA of thyroid nodules with high inadequate risk, for identifying the value of CEUS in thyroid nodule FNA guidance.

## Methods

### Subjects

**Fig. 1:**
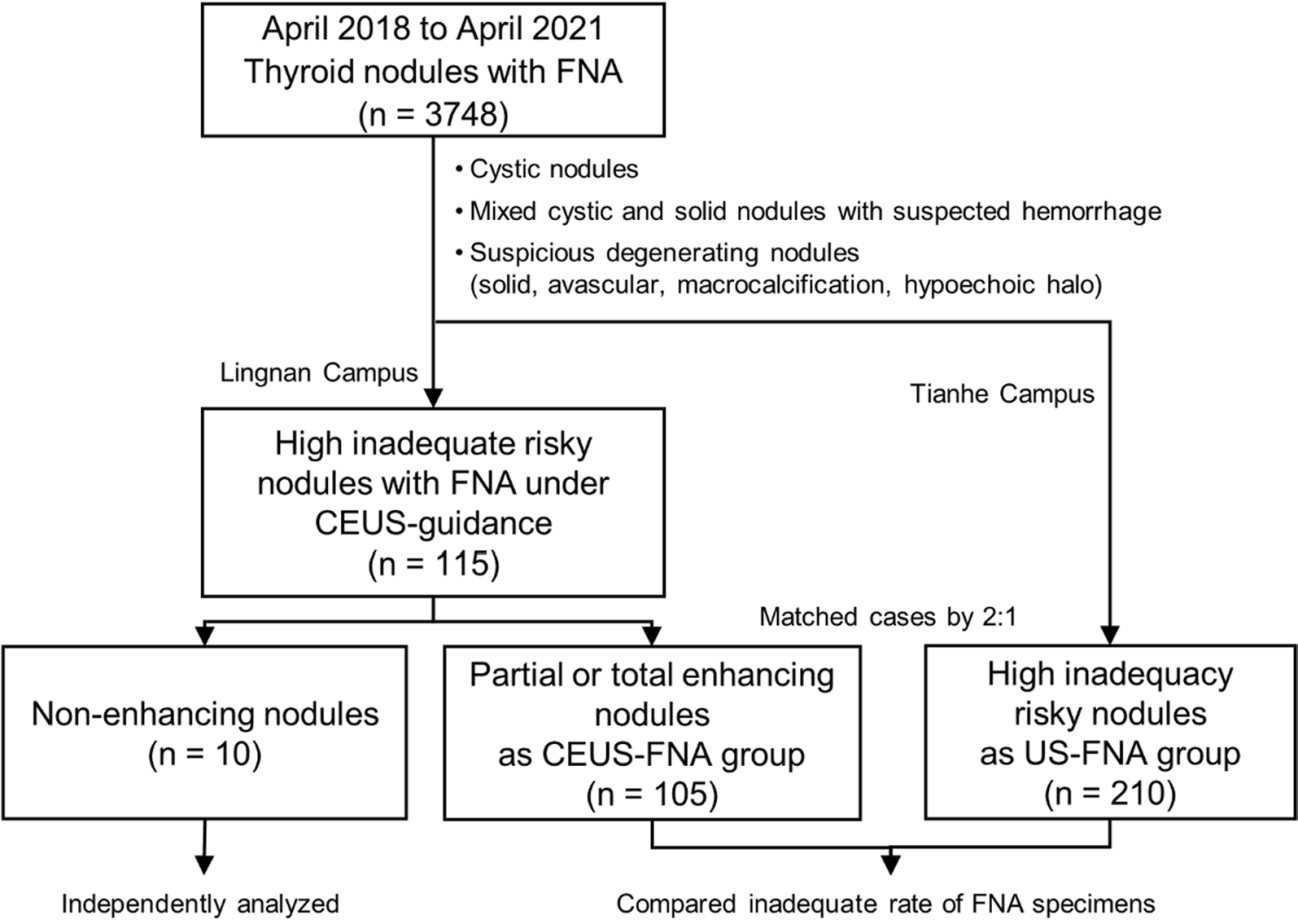
Flow chart of thyroid nodules with FNA enrolled in this study

The present retrospective study was approved by the Ethics Committee of the Third Affiliated Hospital of Sun Yat-sen University. All methods of the manuscript were carried out in accordance with the guidelines issued by the Ethics Committee of the Third Affiliated Hospital of Sun Yat-sen University. The population of the study consisted of 3748 thyroid nodules underwent FNA between April 2018 and April 2021 in two campuses (Lingnan Campus and Tianhe Campus) in our hospital. The inclusion criteria of high inadequacy risk nodules were as follow: cystic nodules, mixed cystic and solid nodules with suspected hemorrhage, or suspicious degeneration nodules (solid, avascular, macrocalcification, hypoechoic halo) [8, 14]. One hundred and fifteen nodules shown high risk of specimen inadequacy during preoperative conventional ultrasonography were enrolled in Lingnan Campus, and the FNAs were carried out by CEUS guidance. Ten of the 115 nodules shown total non-enhancement during CEUS imaging, and would be analyzed independently. The other 105 high inadequate risk thyroid nodules with partial or total enhancement were included in CEUS-FNA group. During the same period, nodules underwent US-guided FNA were included as controls, which were matched to cases according to the criteria of high inadequacy risk nodules in Tianhe Campus, by 2:1 (Fig. 1).

### CEUS- and US-guided FNA

US-guided FNA were performed under the guidance of a clinical US imaging system of Logiq E9 with a 9L linear transducer (GE Medical Systems, USA). Local anesthesia at the puncture site was given (1–3 mL of 1% lidocaine). All FNA cases of the patients were carried out by radiologists in our hospital using 23- or 25-gauge (5 cm in length) needles with real-time US guidance. The needle punctured into the nodule with multiple to-and-fro motions for specimen obtaining. According to the amount of specimen, 3–6 passes were performed for each nodule. Aspirated specimen was injected into a bottle containing liquid-based solution (BD, USA) and transferred to the department of pathology for slide preparing. Papanicolaou and H&E staining were applied for each specimen.

**Fig. 2:**
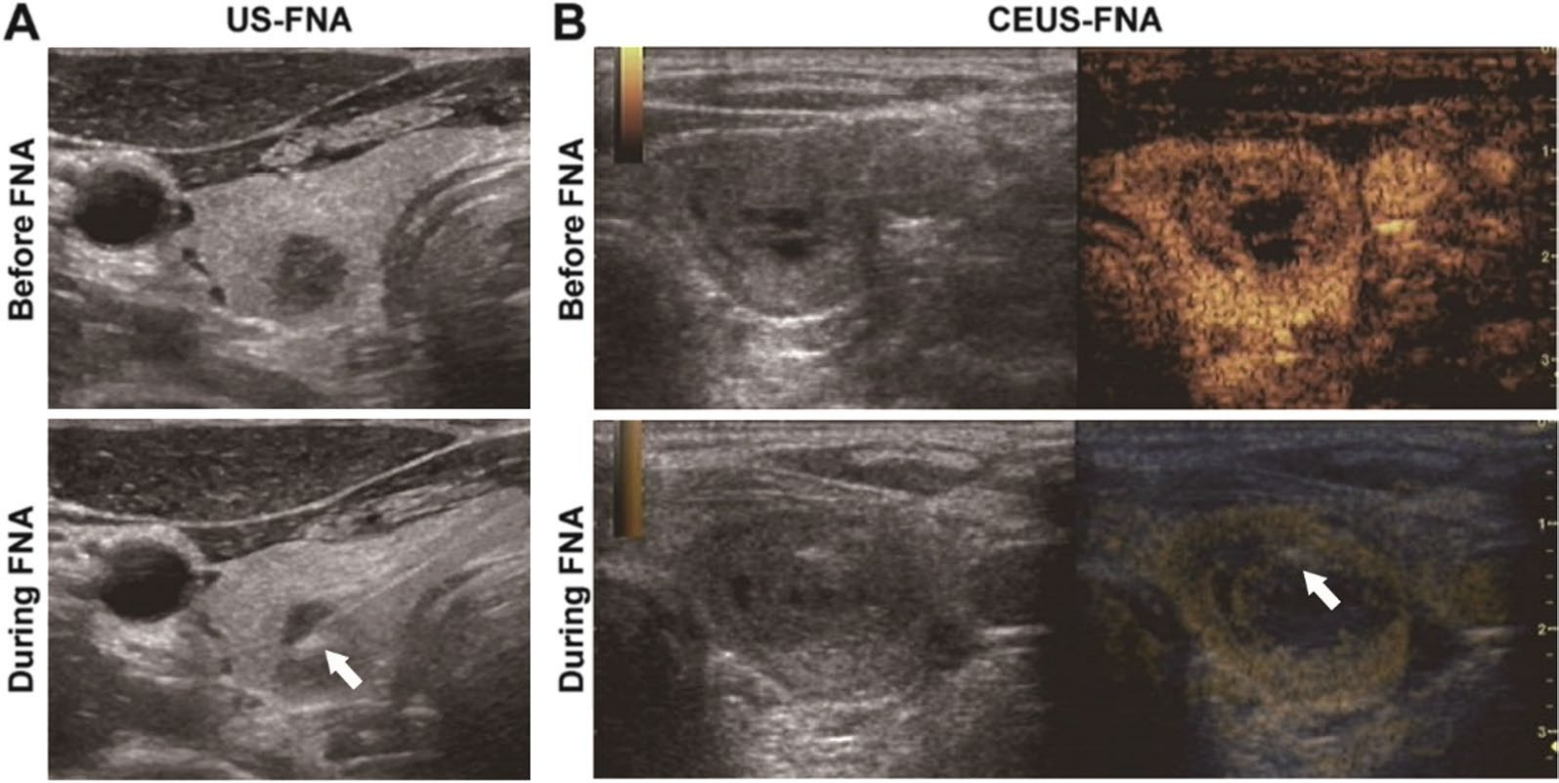
Processes of US- or CEUS-guided thyroid nodule FNA. **A** Ultrasonography guiding FNA of a 6 mm × 7 mm × 8 mm solid nodule in the left thyroid lobe. **B** CEUS visualized the survival tissue of a 21 mm × 15 mm × 23 mm solid nodule in the right lobe of thyroid gland, and guided the needle punching in real-time. The white arrows marked the needle tips

CEUS-guided FNA were performed in Lingnan Campus of our hospital, by using clinical US imaging systems including Logiq E9 with a 9L linear transducer, EPIQ-7 with a L12-5 linear transducer (Philips, Best, Netherlands) or Aplio i800/i900 with a i18LX5 linear transducer (Canon Medical Systems Corporation, Tochigi, Japan). Before the procedure of needle puncturing, CEUS imaging was performed. 2.0 mL of US contrast agent SonoVue (Bracco, Milan, Italy) was bolus applied intravenously, and then 5 mL of normal saline was injected immediately. After comprehensive scanning of the whole nodule, puncture area was confirmed as follow: (1) Solid components with obvious (hyper-, hypo- or iso-) enhancement; (2) Cyst walls with obvious enhancement. If the enhancing area of the nodule was rare or difficult to reach, another bolus of 2.0 mL of SonoVue would be applied for real-time CEUS guidance (Fig. 2). The puncture sites of the 10 total non-enhancing nodules were set to the cyst walls (for cystic nodules) or peripheral zone (for solid or mixed nodules).

### Ultrasonogical characteristics

Ultrasonogical images or videos were retrospectively reviewed by two radiologists (T.Y. with 6 years’ and J.R. with 20 years’ clinical experience in thyroid US examination). Nodule size (largest diameter), location and composition were record. All nodules were classified as cystic (cystic portion > 90%), predominantly cystic (50% < cystic portion ≤ 90%), predominantly solid (10% < cystic portion ≤ 50%) and solid (cystic portion ≤ 10%) [4].

### Cytological classification

All cytological slides were retrospectively read by two cytopathologists (H.L. with 8 years’ and Y.L. with over 10 years’ clinical experience in cytological diagnosis), who were blinded to the US features and puncture guidance during FNA. The cytological results were classified following the Bethesda System for Reporting Thyroid Cytopathology [15]. Specimens less than 6 groups of 10 well-visualized follicular cells or only consisted of cystic contents under microscopy, which were reported as Bethesda Class I, were defined as ‘inadequate’. The other categories from Bethesda Class II to VI were defined as ‘adequate’.

### Statistical analysis

Quantitative data were expressed as mean ± standard deviation or medians with interquartile, and analyzed using a tow-tailed *t*-test or Mann–Whitney *U* test. The categorical variables were analyzed by the chi-square test or Fisher exact test. A *P* value < 0.05 was considered as a statistically significant difference. All statistical analyses were carried out by using SPSS software (version 19, IBM Corp., Armonk, USA).

## Results

**Fig. 3:**
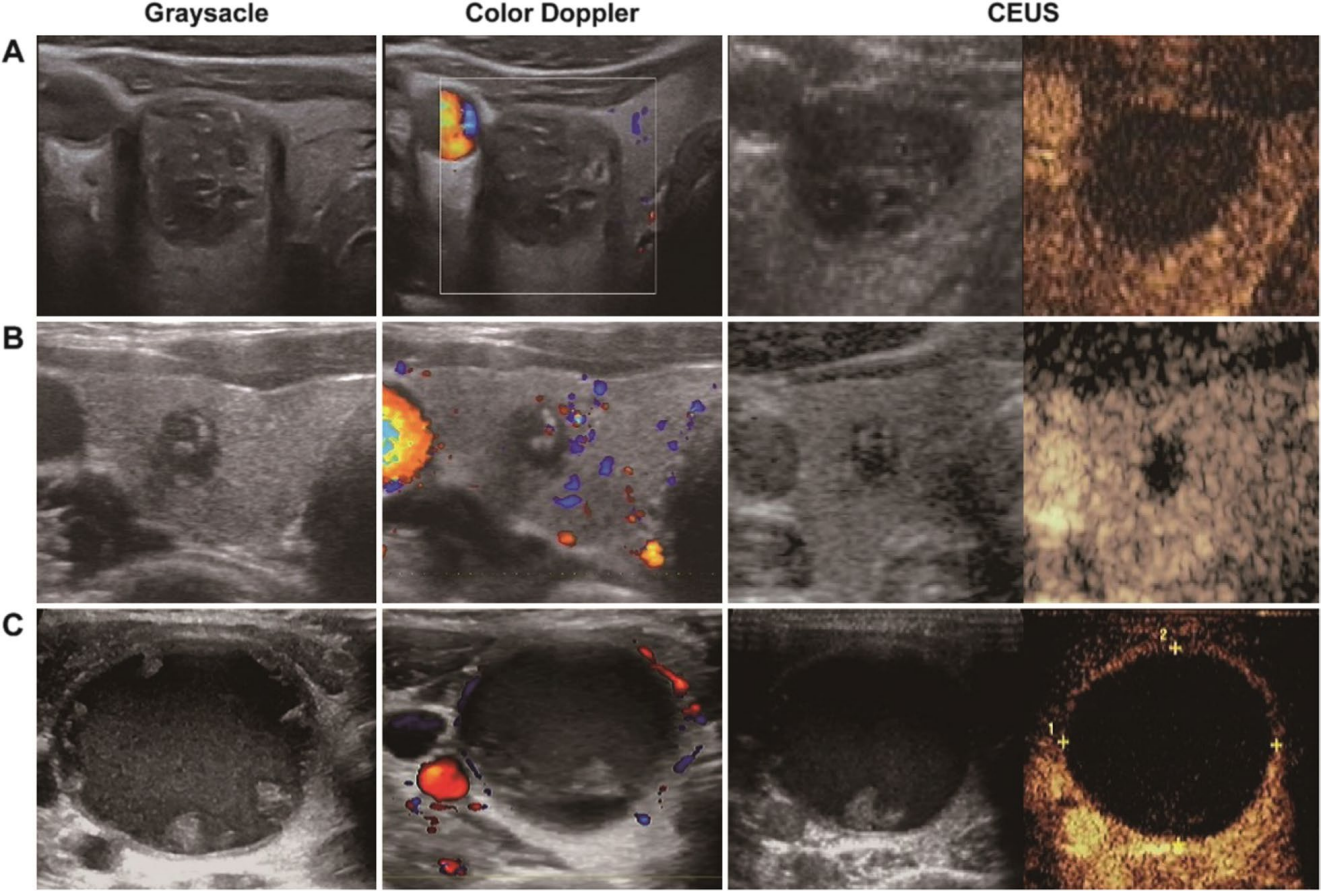
Representative ultrasonography and CEUS appearances of the totally non-enhancing thyroid nodules. **A** A 17 mm × 15 mm × 24 mm solid suspicious degenerating nodule with macro- and microcalcification in right lobe of thyroid of a 54 years old man. **B** A 5 mm × 5 mm × 8 mm very hypoechoic solid nodule with multi-macrocalcification in right lobe of thyroid gland of a 32 years old woman. **C** A cystic large nodule with size of 34 mm × 27 mm × 44 mm in the left thyroid lobe of a 35 years old woman. The solid components in all nodules shown totally non-enhancing during CEUS imaging. Final cytopathologic results were Bethesda class I and classified as inadequate specimen

**Fig. 4:**
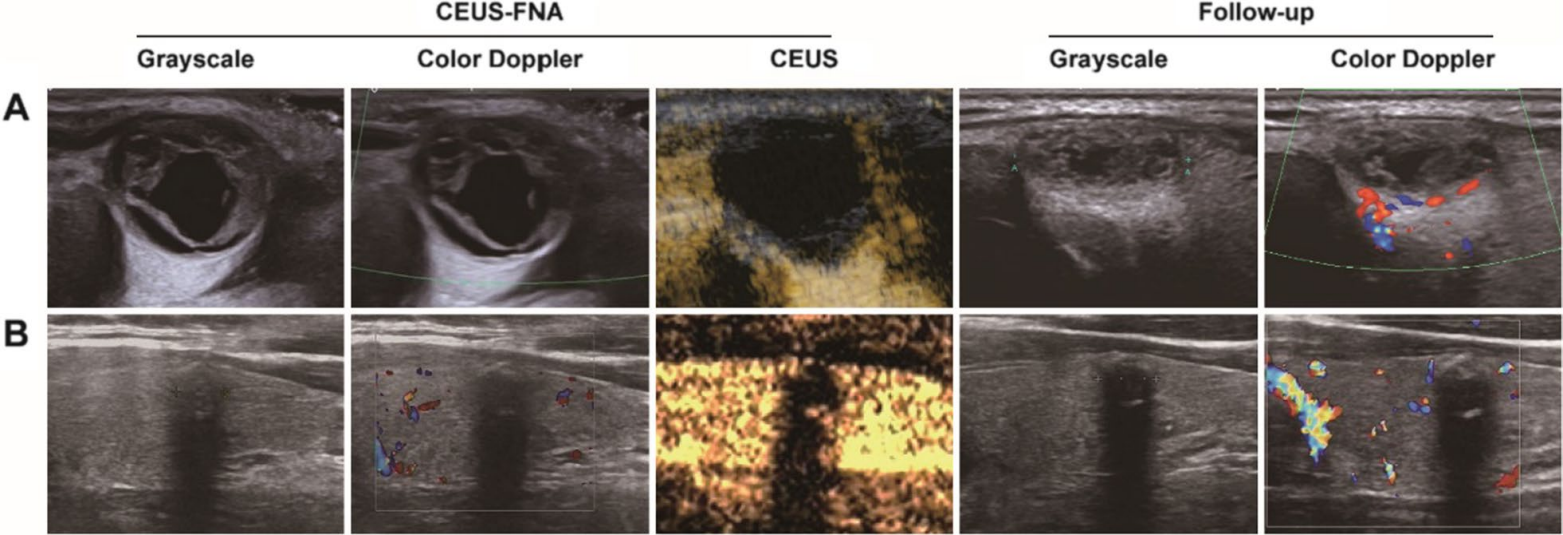
Representative ultrasound features of the totally non-enhancing thyroid nodules with FNA results of inadequate specimen. **A** A 37 years old woman with predominantly cystic nodule in the isthmus of thyroid gland. One month after the CEUS-FNA, ultrasonic follow-up demonstrated the reduction in size and increasing of solid component. The nodule was diagnosed as degenerating nodule. **B** A solid nodule in left thyroid lobe of a 43 years old woman. After 12 months of CEUS-FNA, there was no significant changes in size or ultrasonic feature was found during ultrasonography follow-up

During the study period, the overall cytological inadequate rate of the 3748 thyroid nodules underwent FNA was 6.6%. Ten of the high inadequacy risky thyroid nodules showed completely non-enhancing during CEUS, all of which were cytologically classified as inadequate. Figure 3 illustrated the typical non-enhancing nodules during CEUS. One predominantly cystic and three cystic nodules were cytologically reported few follicular cell clusters in slides without atypia. Size reductions were record of three nodules (one cystic, one predominantly cystic and one solid) during follow-up by ultrasonography (Fig. 4A). Two cystic nodules were clinically diagnosed as cystic benign nodule. One predominantly solid nodule was surgically resected because malignancy nodule was confirmed by CEUS-FNA in the opposite lobe of thyroid gland, and reported as nodular goiter. Three solid nodules were stable in size and morphology during follow-up by ultrasonography, without suspicious neck lymph node detection (Fig. 4B). All of the detail and follow-up of the ten CEUS non-enhancing and specimen inadequate nodules were shown in Table 1.

**Table 1:** Detail and follow-up of the ten CEUS non-enhancing and specimen inadequate thyroid nodules

	Age (year)	Gender	Size (mm × mm × mm)	Location	Composition	Follow-up
1	57	Female	29 × 16 × 32	Right lobe	Cystic	Ultrasonography follow-up 2 months after FNA Nodule size reduced to 22 mm × 11 mm × 27 mm
2	35	Female	34 × 27 × 44	Left lobe	Cystic	Follow-up not done Clinically diagnosed as benign cystic nodule
3	33	Female	26 × 23 × 37	Right lobe	Cystic	Follow-up not done Clinically diagnosed as benign cystic nodule
4	37	Female	17 × 11 × 18	Isthmus	Predominantly cystic	Ultrasonography follow-up 1 months after FNA Nodule size reduced to 13 mm × 5 mm × 15 mm
5	66	Female	4 × 5 × 6	Left lobe	Predominantly solid	Surgically resected (malignancy nodule confirmed in the opposite lobe) Pathological diagnosed as nodular goiter
6	54	Male	17 × 15 × 24	Right lobe	Solid	Ultrasonography follow-up 3 months after FNA Nodule size reduced to 15 mm × 12 mm × 19 mm
7	68	Female	8 × 5 × 10	Right lobe	Solid	Ultrasonography follow-up 12 months after FNA Stable in size and morphology
8	43	Female	6 × 5 × 6	Left lobe	Solid	Ultrasonography follow-up 12 months after FNA Stable in size and morphology
9	61	Female	9 × 7 × 14	Left lobe	Solid	Ultrasonography follow-up 6 months after FNA Stable in size and morphology
10	32	Female	5 × 5 × 8	Right lobe	Solid	Follow-up not done

The characteristics of the partial or total enhancement nodules and matched nodules were showed in Table 2. No significant difference was detected in patient age, gender, nodular size, nodular location and composition between CEUS-FNA and US-FNA groups (*P* > 0.05).

**Table 2:** Characteristics of subjects

Items	CEUS-FNA (n = 105)	US-FNA (n = 210)	*P* value
Age (year)	44.0 ± 14.5	42.3 ± 13.7	0.336
Gender			0.904
Male	22 (27.7%)	45 (22.1%)	
Female	75 (77.3%)	159 (77.9%)	
Size (mm)	24.0 (11.0, 34.0)	23.0 (12.8, 36.0)	0.722
Location			0.987
Right	53 (50.5%)	108 (51.4%)	
Left	46 (43.8%)	90 (42.9%)	
Isthmus	6 (5.7%)	12 (5.7%)	
Composition			0.203
Cystic	27 (25.7%)	64 (30.5%)	
Predominantly Cystic	15 (14.3%)	41 (19.5%)	
Predominantly Solid	18 (17.1%)	40 (19.0%)	
Solid	45 (42.9%)	65 (34.9%)	
Bethesda Class			0.042
I	7 (6.7%)	35 (16.7%)	
II	61 (58.1%)	124 (59.0%)	
III	9 (8.6%)	12 (5.7%)	
IV	8 (7.6%)	5 (2.4%)	
V	14 (13.3%)	22 (10.5%)	
IV	6 (5.7%)	12 (5.7%)	

All data were presented as number of items with percentage in parentheses, excepted age (year) and size (mm)

The details of FNA cytological inadequacies in CEUS-FNA and US-FNA groups, and further stratified comparison were showed in Table 3. According to US-FNA, with increasing nodular cystic portion in the four subgroups, raising inadequate rates were detected (Solid: 9.2%, Predominantly solid: 10.0%, Predominantly cystic: 17.1%, and Cystic of 28.1%; *P* = 0.019).

**Table 3:** Cytological inadequate rates of FNA specimens with CEUS- and US-guidance

	CEUS-FNA (n = 105)	US-FNA (n = 210)	*P* value
Cystic	7.4% (2/25)	28.1% (18/46)	0.029
Predominantly cystic	6.7% (1/14)	17.1% (7/34)	0.428
Predominantly solid	5.6% (1/17)	10.0% (4/36)	0.577
Solid	6.7% (3/42)	9.2% (6/59)	0.630
Total	6.7% (7/98)	16.7% (35/175)	0.014

Data were presented as inadequate rate, with number of inadequate/adequate items in parentheses

**Fig. 5:**
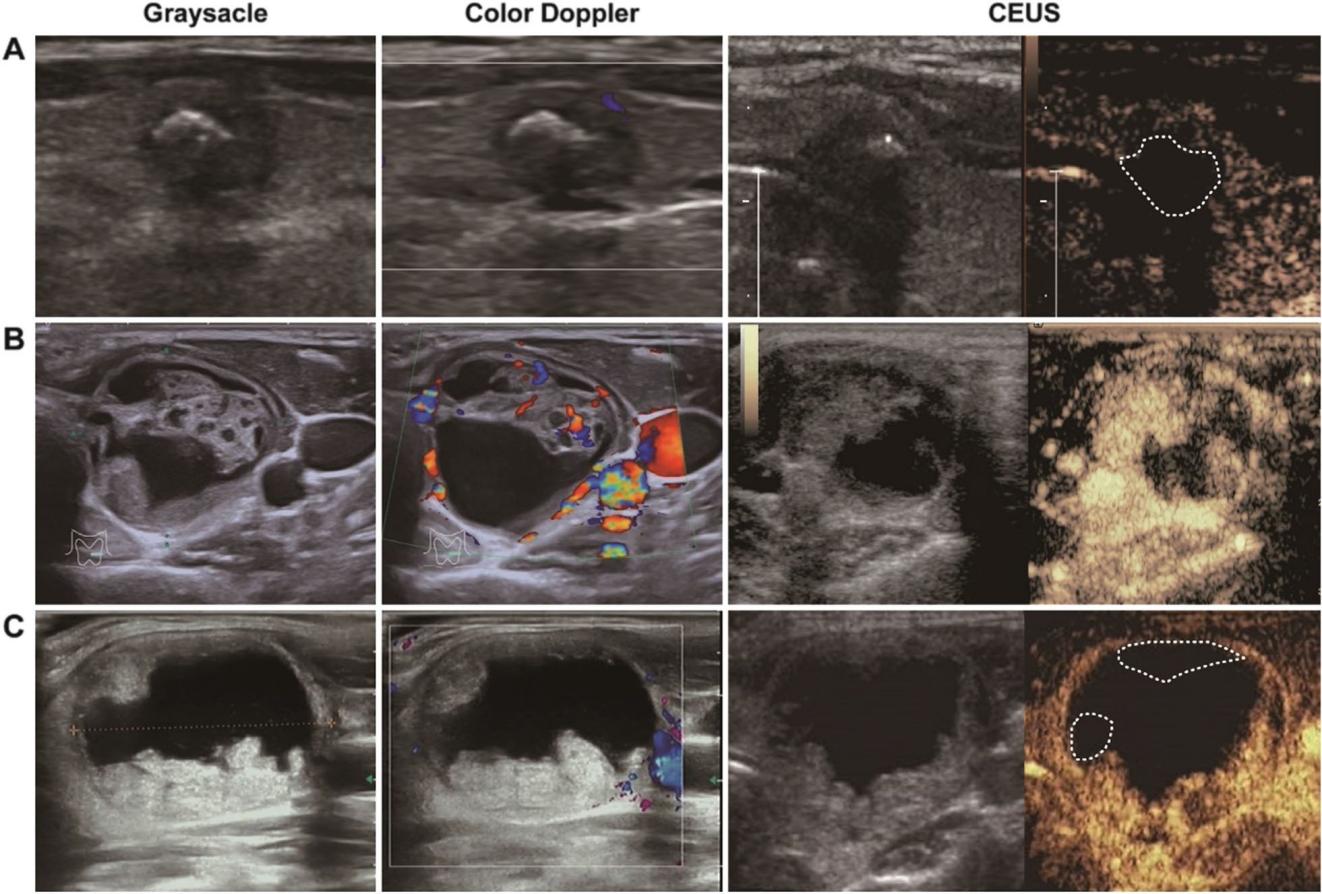
Representative ultrasonography and CEUS appearances of high FNA inadequate risky thyroid nodules. **A** A 6 mm × 5 mm × 11 mm suspicious degenerating nodule with macrocalcification in the isthmus of the thyroid gland from a 41 years old woman. The survival and inactive parts (white dashed line) of the solid nodule were clearly distinguished by CEUS. The cytological category was Bethesda class V. **B** A 25 mm × 20 mm × 29 mm predominantly cystic nodule in the left lobe of thyroid gland of a 36 years old female. Nearly all solid components were active under CEUS. The CEUS-FNA result was classified as Bethesda class II. **C** A predominantly cystic nodule in the right lobe of thyroid gland with size of 32 mm × 24 mm × 34 mm of a 27 years old woman. Small parts of inactive solid components were distinguished by CEUS (white dashed lines). Fine-needle puncturing to the active area resulting in a Bethesda Class II specimen

According to the nodules with high inadequate risk, inadequate rate in CEUS-FNA group was 6.7%, which was significantly lower than 16.7% in US-FNA group (*P* = 0.014), and similar to the 6.6% in overall inadequate rate in our center (*P* = 0.975). Data of the stratified analysis indicated that in cystic subgroup, the inadequate rate of CEUS-FNA was significantly lower than US-FNA (7.4% vs. 28.1%, *P* = 0.029). Furthermore, lower inadequate rates were also observed in CEUS-FNA for predominantly cystic nodules (6.7% in CEUS-FNA vs. 17.1% in US-FNA), predominantly solid nodules (5.6% in CEUS-FNA vs. 10.6% in US-FNA) and solid nodules (6.7% in CEUS-FNA vs. 9.2% in US-FNA), although these differences were not statistically significant (*P* > 0.05). Figure 5 showed the representative CEUS-guiding FNA cases.

## Discussion

US-FNA is known as a reliable and cost-effective method for distinguishing benign and malignant thyroid nodules [16]. The high specimen inadequate rate (3–38%) is still a concern in clinical practices [3]. In the present study, we retrospectively analysis the value of CEUS in FNA guidance for high inadequate risky thyroid nodules. CEUS is an effective real-time imaging modality for observing microvascular perfusion and sensitively detecting viable tissue of thyroid nodules [13].

Thyroid nodules with high inadequate risk have a significantly higher inadequate rate after US-FNA in our study, comparing with the overall 3748 nodules in our center (16.7% vs. 6.6%, *P* < 0.001) or some other centers [17]. They have some common US features, such as high cystic composition, mixed cystic and solid nodules with hemorrhage, or suspicious degenerating nodules [4, 6]. For instance, the inadequate rates of US-FNA in cystic, predominantly cystic, predominantly solid and solid subgroups are 28.1%, 17.1%, 10.0% and 9.2% (*P* = 0.019), which indicating that the amount of cystic component is an important factor affecting specimen adequacy. This result is consistent with reported literature [4, 18]. In addition, for the predominantly solid and suspicious degenerating solid nodules, old hemorrhage, fibrosis, infarction, and calcification are common pathologically [19]. All these changes are difficult to distinguish from viable tissue by grayscale US and even color Doppler US [20].

In comparison, our results indicate that the total inadequate rate of FNA with CEUS guidance is significantly lower than US guidance in thyroid nodules with high inadequate risk (6.7% vs. 16.7%). From the stratification data, inadequate rates of the nodules of with CEUS-FNA are 7.4% in cystic, 6.7% in predominantly cystic, 5.6% in predominantly solid and 6.7% in solid subgroups (*P* = 0.996). CEUS guidance eliminates the effect of cystic components to FNA specimen adequacy, by reducing the inadequate rate of cystic thyroid nodules (7.4% vs. 28.1%, *P* = 0.029). Moreover, our results also show that, the adequacies with CEUS guidance also pronounced in the other subgroups: predominantly cystic nodules (6.7% vs. 17.1%), predominantly solid nodules (5.6% vs. 10.0%) and solid nodules (6.7% vs. 9.2%), even though there are no significant differences. All these results indicate that CEUS elevates the specimen adequacy of FNA in high-risk thyroid nodule, even to the level of overall average.

We hypothesize the high cytological adequacy of CEUS-FNA relates to the viable tissue targeted puncture. For one hand, CEUS accurately distinguishes viable tissues from inactive, and locates the viable tissues within nodules [21, 22]. Viable tissues, including follicular tissue, granulation tissue, and tumor tissue are shown certain degrees of contrast enhancements under CEUS. However, inactive tissue, such as old hemorrhage, fibrosis, infarction, and calcification generally shown nonenhancement. As a result, viable tissues are easily visualized and located in thyroid nodules by CEUS. For another hand, CEUS provides precisely guidance in real-time for nodule puncturing. As viable tissue shown continuously enhancement in thyroid nodules, CEUS imaging is capable for guiding the fine needle to the targeted tissue in real-time, even though viable tissues are rare or macrocalcifications are present.

From another point of view, we have found 10 completely non-enhancing nodules before the FNA procedure and none of them is reported as cytological adequate. The postoperative pathology and follow-up ultrasonography have shown the non-enhancing nodules are benign or no further malignant features. Li et al. also reported in a literature the nonenhancement nodules are benign [23]. As CEUS show total non-enhancement of a thyroid nodule, representing that no viable tissue was found within the nodule, no or very few follicular epithelial cells will be achieved after FNA. This result suggests that FNA is not recommended in completely non-enhancing nodules under CEUS because viable tissues are rare. However, PTC with severe fibrosis and calcifications occasionally show US pattern of heavily calcified nodule or entirely calcified nodule may also show non-enhancement of CEUS [24]. For those thyroid nodules with high clinical suspicion or patient with extremely anxious, core-needle biopsy is more effective than FNA. Our recommendation may avoid a certain amount of unnecessary FNAs.

There are several limitations in the present study. First, the retrospective design may lead to selective bias. Second, unequal distribution of samples may reduce the statistical power, particularly for subgroup analyses. Third, as FNA is an experience-dependent procedure, different operators may obtain various specimen adequate rates. However, inter-operator variability was not carried out in the present study.

## Conclusion

CEUS can improve the specimen adequacy of FNA in high inadequate risk nodules from two aspects: (1) by avoiding unnecessary FNAs; (2) by accurately locating the survival tissue and precise guidance in real-time. According to our results, CEUS is a recommend imaging modality for FNA guidance of high inadequate risk thyroid nodules.

## Data Availability

The datasets generated during and analysed during the current study are not publicly available due to their containing information that could compromise the privacy of research participants but are available from the corresponding author on reasonable request.

## Abbreviations

CEUS: Contrast-enhanced ultrasound
FNA: Fine-needle aspiration
CEUS-FNA: CEUS-guided FNA
US-FNA: Ultrasound guided FNA
